# Effects of Flash Evaporation Conditions on the Quality of UHT Milk by Changing the Dissolved Oxygen Content in Milk

**DOI:** 10.3390/foods11152371

**Published:** 2022-08-08

**Authors:** Shiyao Jiang, Wenjing Luo, Qiuqi Peng, Zhengyan Wu, Hongbo Li, Hongjuan Li, Jinghua Yu

**Affiliations:** Key Laboratory of Food Nutrition and Safety, Ministry of Education, College of Food Science and Engineering, Tianjin University of Science and Technology, No. 29, No. 13 Ave., TEDA, Tianjin 300457, China

**Keywords:** flash deoxygenation, stability, oxidation, Maillard reaction, color, flavor

## Abstract

This study assessed the impact of reducing dissolved oxygen (DO) content on the quality of UHT milk using a flash deoxygenation treatment. Flash deoxygenation was designed based on preheated milk reaching boiling early under low-pressure conditions to remove DO from the milk. Two parameters were designed for flash deoxygenation: preheating temperature 65 °C, −0.08 Mpa, and 70 °C, −0.06 Mpa. The flash conditions were applied to two UHT sterilization conditions (135 °C for 10 s and 145 °C for 5 s). After deoxygenation, the total oxidation (TOTOX) value of UHT milk was reduced by 1.4~1.71, and the protein carbonyl (PC) value was reduced by 1.15~1.52 nmol/mg of protein. The maximum inhibition rates of furusine and 5-HMF were 33.23 ± 1.72% and 25.43 ± 3.14%, respectively. The particle size was reduced by 0.141~0.178 μm. The ketones and stale aldehydes causing oxidized taste in the UHT milk were significantly reduced. This study showed that the oxidation and Maillard reactions of UHT milk were significantly inhibited, stability was improved, and the content of undesirable volatile flavor substances was reduced after flash deoxygenation. Therefore, reducing DO content was beneficial to improving the quality of UHT milk.

## 1. Introduction

Ultra-high-temperature (UHT) processing of milk can effectively inactivate pathogenic microorganisms and enzymes and extend shelf life. However, UHT sterilization has certain adverse effects on milk. The most notable of which are protein and lipid oxidation, Maillard reactions, and lactose isomerization [1,2]. According to studies, the oxidation level of milk is strongly linked to various chemical events that degrade milk quality. Therefore, in recent years, the removal of dissolved oxygen (DO) from raw milk before UHT treatment was adopted in dairy companies to improve the quality of UHT milk.

DO in raw cow’s milk mainly comes from the collection of milk. Milk rapidly absorbs surrounding oxygen when it leaves the breast milk, and within a few hours between collecting raw milk and storing it in a tank at 5 °C, its DO content increases about fivefold [3]. This absorbed DO is not only present in milk as oxygen molecules (O_2_) but also as diradical triplet oxygen (^3^O_2_) and singlet oxygen (^1^O_2_). O_2_ and ^3^O_2_ are the most abundant and stable forms of oxygen in milk [4].

DO plays a crucial role in the quality of UHT milk processing. Thus far, the effect of DO on the quality of UHT milk consists mainly of the following aspects. Firstly, DO produces the oxidation of vitamins in milk, such as vitamin C and folic acid, during the heating process. Removal of DO from milk before heat treatment was found to be apparently essential to protect ascorbic acid and folic acid in milk by N_2_ replacement of DO in pasteurized milk to reduce DO levels [5]. In addition to the effect on vitamins, lipids are also affected by the DO content [6]. Lipid oxidation was inhibited entirely in sterilized milk beverages saturated with N_2_ or N_2_/H_2_ and improved the antioxidant properties of dairy products during storage [7]. Secondly, the Maillard reaction is also an essential factor affecting the quality of UHT milk. As previous experiments have shown, olefinic diols are key intermediates during the early stages of the Maillard reaction. DO can be removed by vacuum treatment or bubbling with N_2_, avoiding the oxidative cleavage of the olefinic diols. In addition, Aminoreductone (AR) is one of the products of the Maillard reaction. The decrease in DO content in raw milk before heating will inhibit the oxidative degradation of AR during the heating process to maintain a high AR concentration after heating, which is beneficial to maintaining the quality of commercial milk [8]. In terms of color, the DO level also plays a role in milk. For example, ascorbic acid in milk is oxidized to dehydroascorbic acid with further formation of decomposition products such as furfural with a brown color [9]. Finally, changes in milk flavor are also related to DO. Some aged flavor volatiles were significantly reduced by reducing the DO content in UHT milk using a deoxygenated membrane [10]. Therefore, reducing DO content will have a positive impact on UHT milk quality.

At present, the main means of deoxygenation commonly used in the industry for milk beverages are gas sparging, flash deoxygenation, and membrane deoxygenation. Nevertheless, the disadvantage of gas sparging is that flavor volatile components are removed and lost to the atmosphere [11], and the disadvantage of membrane deoxygenation is the growth of bacteria [12]. The flash deoxygenation technology is the most widely used in the processing of dairy products, and its main purpose is to remove the peculiar smell in milk and increase the content of constituent substances in the milk. However, the effect of flash deoxygenation on the quality of UHT milk has not been reported. In this study, two commonly flash deoxidation conditions (65 °C, −0.08 MPa; 70 °C, −0.06 MPa) were used to deoxidize milk, and two different UHT sterilization conditions (135 °C, 10 s; 145 °C, 5 s) were selected. The unflashed UHT milk was selected as the blank group for comparison, and the effects of DO content changes on the stability, fat and protein oxidation, Maillard reaction, color, and flavor of UHT milk were observed.

## 2. Materials and Methods

### 2.1. Sample Preparation

Fresh raw bovine milk was obtained from a local dairy farm (Hebei, Shijiazhuang, China). Before all treatments, the milk was standardized to contain 3.03% protein, 2.85% fat, 4.05% lactose, and 11.24% dry matter. Fresh raw milk was preheated to 55 °C and then homogenized twice at a homogenization pressure of 20 MPa using a two-stage homogenizer (model AH-BASIC II, Triowin, Shanghai, China; rated flow: 20 L/h). Finally, the samples were sterilized by UHT at 135 °C for 10 s and 145 °C for 5 s.

### 2.2. Flash Deoxidation

**Figure 1:**
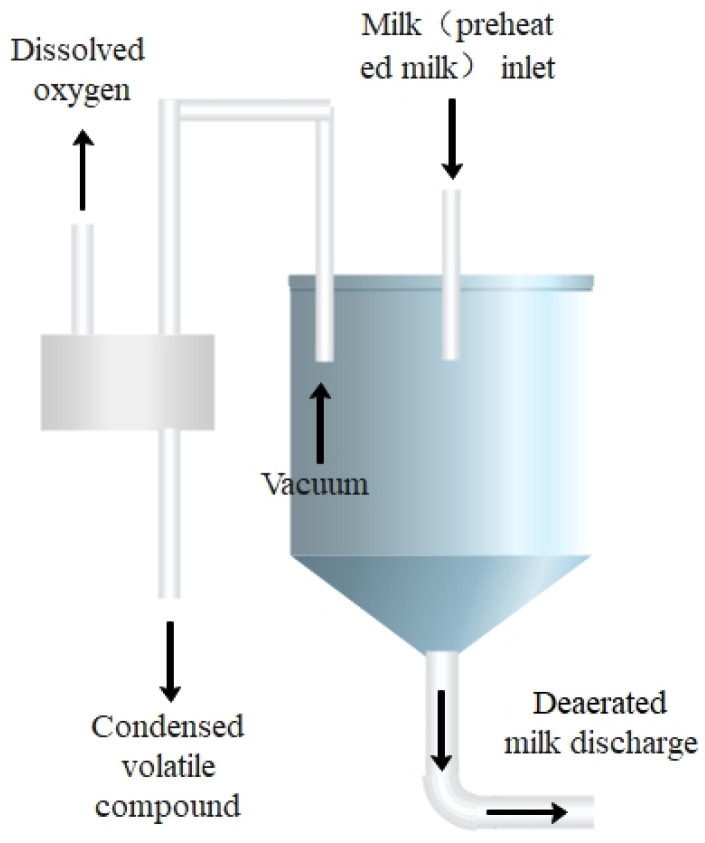
Flash deoxidation equipment.

The principle of flash deoxygenation is that the preheated milk will reach boiling early under low-pressure conditions, at which time not only the DO in the milk will be removed, but also the undesirable flavor substances will be removed. Figure 1 depicts the flash equipment used in this experiment. The preheated milk is put in the flash tank, and the low-pressure environment of the flash tank causes the hot milk to boil, removing the DO from the milk. Flash deoxygenation is performed before the homogenization of milk, and the two most commonly used flash conditions in the dairy production process are selected as 65 °C, −0.08 MPa, and 70 °C, −0.06 MPa, respectively.

### 2.3. Determination of DO

The dissolved oxygen content in milk was determined using a dissolved oxygen meter (LE Sensors, Mettler, Switzerland) based on the principle of redox. The dissolved oxygen content was determined by adding 40 mL of milk to the dissolved oxygen measuring bottle.

### 2.4. Determination of Milk Fraction

The fat and protein contents of milk were determined using the Rhodes–Gottry and the Kjeldahl methods, respectively. The lactose content in milk was determined using a lactose kit (Jiancheng Institute of Biological Engineering, Nanjing, China). The dry matter content was determined by high-temperature drying.

### 2.5. Turbiscan Measurement

The stable direction of the sample was determined by Turbiscan Lab (Turbiscan Easy Soft, Formulation, Smart Scientific Analysis, Toulouse, France) under the gravity force. The backscattering intensity as height function was measured under the near-infrared light source of 880 nm. At room temperature, it was scanned every 60 min for 7 h. The TSI value of the sample can be directly obtained.

### 2.6. Particle Size Measurements

The size distributions were measured in the whole milk by laser light scattering using a laser particle size distribution meter (Bettersize2600, Bettersize, Guangdong, China). The refractive indices used were 1.52 for milk at 466 nm, and 1.33 for water. The experiments were conducted at room temperature. First, 1 mL milk samples were diluted with distilled water 10 times, then 1 mL diluted milk samples were added with 2 mL 35 mmol/L EDTA/NaOH (pH = 7) solution to dissociate casein micelles, and then 1–2 drops of 10% SDS solution were added to disperse and aggregate fat spheres. During the test, the sample addition should ensure that the shading rate of the laser particle size analyzer was between 10% and 11% (optimal conditions for particle-size measurements with this apparatus). Volume-surface mean diameter (D[3,2]) was calculated by the instrument software as follows:

(1) $$D\left[3,2\right]=\frac{\sum \left(n_{i}\times d_{i}{}^{3}\right)}{\sum (n_{i}\times d_{i}{}^{2})}$$

where n_i_ is the volume of particles in a size class of diameter *d*_i_.

### 2.7. Measurements of Primary and Secondary Lipid Oxidation Products

Peroxide value (PV) was determined using a modified method by Shantha and Decker [13]. Then, 0.3 mL of sample was mixed with 1.5 mL of isooctane/2-propanol (3:2, *v*/*v*), vortexed for 10 s each. After being centrifuged at 2000× *g* for 5 min, 0.2 mL of the upper clarified solution was aspirated and mixed with 2.8 mL of methanol/1-butanol (2:1, *v*/*v*), and 0.2 mL of isooctane was mixed with 2.8 mL of methanol/1-butanol (2:1, *v*/*v*) for the blank group, and then 15 μL of KSCN solution (3.94 mol/L) and 15 μL of FeCl_2_ solution (0.072 mol/L) were added and mixed well. The reaction was carried out for 20 min at room temperature and protected from light. Then, 200 μL was taken on an enzyme standard plate, and the absorbance was measured at 510 nm by a UV-1601 UV–visible spectrophotometer (Shimadzu Corp., Columbia, MD, USA). Each measurement was carried out three times. PV (expressed as mmol/L sample) was quantified using a cumene hydroperoxide standard curve (5, 10, 50, 100, 500, and 1000 mol/L in methanol), and calculated using the equation:

(2) $$\mathrm{PV}=\frac{\left(A-b\right)\times 1.5\times 0.6}{a\times 0.3\times 1000}$$

where *A* is the absorbance of the sample against blank. *a* and *b* are slope and y-intercept obtained from the standard curve, respectively.

ρara-Anisidine value (ρ-AnV) was determined according to the AOCS Official Method Cd 18–90 (AOCS, 2009) [14]. Then, 2 mL of sample was added to a 25 mL volumetric flask and made up to volume with isooctane. After mixing and shaking well, the sample was transferred to a 50 mL centrifuge tube and vortexed for 10 s each. After centrifugation at 5000× *g* for 10 min, against isooctane as a blank. The absorbance A_1_ was measured at 350 nm by taking 2 mL of the upper clarified solution. Then, 5 mL of the supernatant was then transferred to a 10 mL test tube, 1 mL of anisidine solution (0.25%, dissolved in glacial acetic acid, *w*/*v*) was added, vortexed for 10 s each, and the reaction was carried out at 20 °C for 10 min. Then, 200 μL of the sample was taken on the enzyme standard plate and the absorbance A_2_ was measured at 350 nm against isooctane containing p-anisidine as a blank. ρ-AnV was calculated as follows:

(3) $$\rho -\mathrm{AnV}=\frac{25\times \left(1.2\times A_{2}-A_{1}\right)}{2}$$

The total oxidation (TOTOX) value was calculated as:

TOTOX = 2 × POV + ρ-AnV(4)

### 2.8. Determination of Protein Carbonyl (PC)

The procedure was adapted from previously reported methods [15,16]. Equal amounts of aqueous buttermilk solution (1 mg protein content) and 1 mL of 10 mM 2,4-dinitrophenylhydrazine (DNPH) hydrochloric acid solution (2 mol/L HCl concentration) were incubated for 30 min at room temperature (25 °C). 10% (*w*/*v*) TCA was added to precipitate the proteins in the milk solution and recovered by centrifugation for 5 min at 7500× *g* (Micromax RF centrifuge, Xiangyi Corp, Hunan, China). The protein precipitate was washed with 1 mL of ethanol/ethyl acetate (1:1, *v*/*v*) to remove the free DNPH reagent and then redissolved in 1 mL of 6 M guanidine hydrochloride (pH = 2.3). Protein carbonyls were determined by UV spectrophotometry (ε_370 nm_ = 2.2 × 104 M^−1^ cm^−1^, where ε = molar absorptivity). The results were expressed as nanomoles carbonyl per mg of protein, and each assay was repeated four times. Protein concentration in bovine milk was determined using the BCA kit.

### 2.9. Dityrosine Quantification

The aqueous solution of milk (protein content of 1 mg) was precipitated with 10% (*w*/*v*) TCA solution and recovered by centrifugation for 5 min at 7500× *g* (Micromax RF centrifuge, International Equipment Company). The recovered protein precipitate was redissolved in 1 mL of 6 M guanidine hydrochloride (pH = 7.0). The dityrosine in the protein solution was measured by fluorometric analysis. Fluorescence spectrophotometry (SLM/Aminco, American Instrument Company, Urbana, IL, USA) was performed with a xenon arc lamp using a 5 nm bandwidth. The formation of dityrosine was monitored by the increase in fluorescence using excitation and emission wavelengths of 315 and 410 nm, respectively, as reported previously [17]. Dityrosine was calculated from the ratio of emission intensities 410 nm and 350 nm and expressed as arbitrary fluorescence units. Each assay was repeated four times.

### 2.10. Determination of Lactulose

Next, 5 mL of milk was centrifuged and defatted at 4 °C for 10 min at 8000× *g*, and the centrifuged emulsion was recovered. The proteins in the emulsion were precipitated by adding 6 mL of anhydrous ethanol, and the recovered emulsion was heated to evaporate the ethanol and re-solubilized with water, and then the sample was filtered through a 0.22 μm filter membrane before HPLC analysis. The HPLC analysis was performed according to Peng Liu et al. [18]. Using an Agilent-1260 liquid chromatographic system (Agilent, America) coupled with a G1311C series controller pump degassing device, RID-10A differential refractive index detector system, a G1329B autosampler injector, and an Aminex HPX-87H Column (5 μm) chromatographic column (7.8 mm × 300 mm). The detection temperature was 55 °C, and the injection volume was 10 μL. The mobile phase was 5 mmol/L H_2_SO_4_. The Chemstation system was used for data acquisition. Lactulose concentrations were calculated by establishing calibration curves in the range of 0.1 and 300 μg/mL (0.1, 1, 10, 20, 100 and 300 μg/mL).

### 2.11. Determination of Furosine

Next, 2 mL of the sample was mixed with 6 mL of 10.6 mol/L HCL solution and then placed in an oven at 110 °C for 18 h. After heating for about 1 h, the sample was removed to cool to room temperature and then shaken once. It was removed and cooled after waiting for the end of hydrolysis, then filtered with a 0.22 μm PES membrane (Pall Corp, New York, NY, USA). Taking 2.00 mL of the hydrolysis solution to determine the protein content in the hydrolysis solution by the Kjeldahl method, 1 mL of the hydrolysis solution mixed with 5.00 mL 0.1% trifluoroacetic acid solution was passed through a 0.45 μm aqueous phase filter membrane for determination by HPLC. The HPLC was performed using an Agilent-1260 liquid chromatography system (Agilent, America) with a G1311C series controller pump degassing device, a G1315D series photodiode array detection system, a G1329B autosampler injector, and an Agilent Eclipse XDB-C18 (5 μm) column (4.6 mm × 250 mm). The detection temperature was 35 °C and the injection volume was 20 μL. The detection wavelength of furosine was 280 nm, and the flow rate was 0.5 mL/min. Acetonitrile (B) and 0.1% trifluoroacetic acid aqueous solution (A) were used as mobile phases. The Chemstation system was used for data acquisition. The concentration of furosine was calculated by establishing calibration curves in the range of 0.5 and 50 μg/mL (0.5, 1.0, 5.0, 10.0, 15.0, 20.0, 25.00, and 50.00 μg/mL).

### 2.12. Determination of 5-HMF

Next, 10 mL of the sample was added to 5 mL 0.15 mol/L oxalic acid in a beaker. Additionally, then, the beaker was placed in a boiling water bath for 25 min after being covered with plastic wrap film. After cooling to room temperature, 2 mL of 40% (*w*/*v*) TCA was added and centrifuged (8000× *g*, 10 min), and then the supernatant was collected and filtered (0.22 μm) before HPLC analysis. The separation was performed using an Agilent-1260 liquid chromatography system (Agilent, America) coupled with a G1311C series controller pump degassing device, a G1315D series photodiode array detection system, a G1329B autosampler injector, and an Agilent Eclipse XDB-C18 (5 μm) chromatographic column (4.6 mm × 250 mm). The detection temperature was 30 °C, and the injection volume was 20 μL. The detection wavelength of HMF was 284 nm, and the flow rate was 0.6 mL/min. Acetonitrile (B) and water (A) (10:90 *v*/*v*) were utilized as mobile phases. The Chemstation system was used for data acquisition. The concentration of 5-HMF was calculated by establishing calibration curves in the range of 0.1 and 300 μg/mL (0.1, 1, 10, 20, 100, and 300 μg/mL).

### 2.13. Determination of Melanoids

Next, 5 mL of the samples were centrifuged at 8000× *g* for 10 min at 4 °C. 1 mL of skimmed milk sample, 20 mL of PBS buffer solution (pH = 8), and 15 mL of 90% ethanol solution were placed in a 50 mL centrifuge tube and sonicated in an ultrasonicator at 40 Hz for 0.5 h. After sonication, the samples were centrifuged again at 3000× *g* for 10 min. After centrifugation, the absorbance of the upper clarified solution was measured at 420 nm, and the higher the absorbance, the higher the content of melanoids.

### 2.14. Determination of Color

The color of the samples was measured using High-Quality Colorimeter NR110 (Shenzhen ThreeNH Technology Co., Ltd., Shenzhen, China). L* (brightness or luminance: L* = 0 black and L*= 100 white), a* (red or green components: −a* = greenness and + a* = redness), and b* (yellow or blue components: −b* = blue and + b* = yellow) were determined parameters. To evaluate color change, *BI* was calculated using the following equation:

(5) $$BI=\left[\frac{0.31\times \left(a^{*}+1.75\times L^{*}\right)}{5.645\times L^{*}+a^{*}-3.012\times b^{*}}\right]/0.172$$

### 2.15. Determination of Flavor

Then, 5 mL sample and 1 g NaCl were mixed in a standard 20 mL headspace vial with a polytetrafluoroethylene (PTFE) septum, which was stirred magnetically at 60 °C for 15 min. At the end of the equilibration phase, the SPME needle (75 µm Carboxend/PDMS) was inserted into the flask through the septum and left exposed in the headspace for 30 min. The SPME needle was then retracted and reopened in the injection port of the GC-MS (Shimadzu Co., Ltd., kyoto, Japan) and heated to 250 °C for 2 min to release the collected volatile components at the beginning of the column. The column used was an Agilent 1909/S capillary column (30 m × 0.25 mm inner diameter: coating thickness 0.25 µm). The carrier gas was He at a flow rate of 2 mL·min^−1^ and a linear velocity of 40 cm·s^−1^ split 1:10; ionization: EI 70 eV. The column temperature was maintained at 40 °C for 3 min, then increased from 40 °C to 180 °C at a rate of 5 °C min^−1^ and maintained at 180 °C for 1 min, then increased from 180 °C to 250 °C at a rate of 7 °C min^−1^ and maintained at 250 °C for 5 min.

### 2.16. Statistical Analysis

All analyses were performed in triplicate, and data were expressed as mean ± Standard Deviation (SD). Statistical significance of differences between samples was analyzed by ANOVA in SPSS statistics 18 software (*p* < 0.05). The data figure was drawn using GraphPad Prism 8.0 (H.J. Motulsky, Prism 7 Statistics Guide, GraphPad Software Inc., San Diego, CA, USA).

## 3. Result and Discussion

### 3.1. Changes in Milk Fraction

The principle of flash evaporation is to transport the preheated milk to the vacuum environment and reach a low boiling point state [19]. The evaporation of milk moisture was achieved to increase the dry matter content of milk. Table 1 shows that each component of the flash-treated milk significantly increased (*p* < 0.05) and the DO content in UHT milk after flash evaporation significantly reduced (*p* < 0.05). Flash deoxygenation is due to the sudden decrease in pressure that preheated milk undergoes as it enters the deaeration tank, producing a practically instantaneous separation [4]. UHT sterilization of milk could also reduce DO content because the DO content in milk can be excreted under heating conditions. Oxygen removal rate from raw milk was 61.09% and 34.13% for flash conditions of 65 °C, −0.08 MPa, and 70 °C, −0.06 MPa, respectively.

### 3.2. Effect of Flash Deoxidation on the Stability of UHT Milk

**Figure 2:**
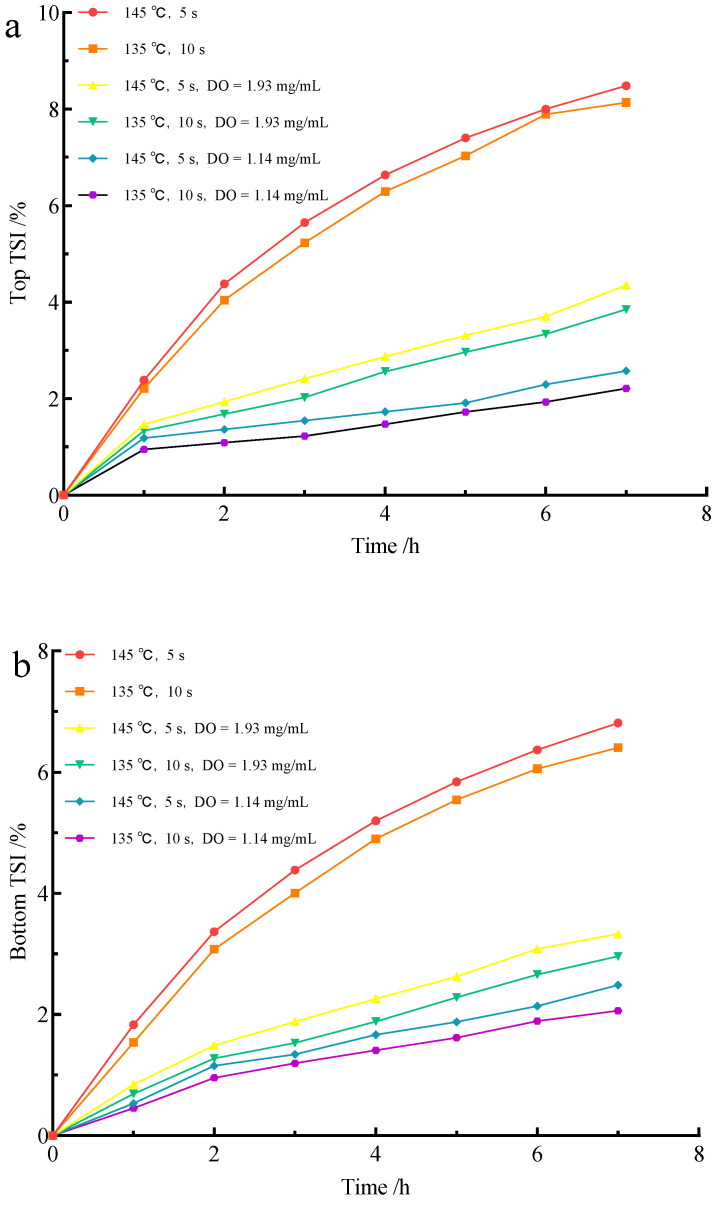
Effect of flash deoxygenation on top (**a**) and bottom (**b**) stability of UHT milk.

TSI is a parameter used to assess the stability of emulsions, and the higher the TSI value, the more unstable the system is [20]. Turbiscan analyzed the changes in the top stability of milk by measuring the changes in background scattered light at the top of the milk, where the creaming was the key to achieving the top stability of milk. As shown in Figure 2a, the top TSI of milk was significantly reduced after flash deoxygenation (*p* < 0.05), which indicated that deoxygenation could effectively reduce the negative effect of UHT sterilization condition on fat stability. This phenomenon may be caused by the lower DO content that facilitates the maintenance of MFGM stability, thus preventing MFG aggregation from occurring. In addition to maintaining the stability of MFGM, the effect of reduced DO content on lipid oxidation is one of the reasons for improving the stability of milk fat [21]. When DO continued to decrease, the stability of milk fat was improved. However, the effect was not obvious (*p* > 0.05), which may be related to the effect of sterilization conditions on accelerating the milk fat floating [22].

Additionally, the stability of the milk bottom was analyzed by measuring the change in background scattered light at the bottom of the milk, where protein precipitation was the key. Figure 2b shows that the TSI at the bottom of the UHT milk was significantly lower (*p* < 0.05) after flash evaporation, indicating that protein precipitation in milk was reduced after deoxygenation. The main causes of protein precipitation in milk are protein denaturation and oxidation. The DO content is positively correlated with protein oxidation [23]. In addition, DO can be adsorbed to surrounding proteins as re-propagation to form larger protein aggregates. However, the reduction in DO does not completely eliminate protein precipitation because UHT treatment has denatured and precipitated some of the proteins [24].

**Figure 3:**
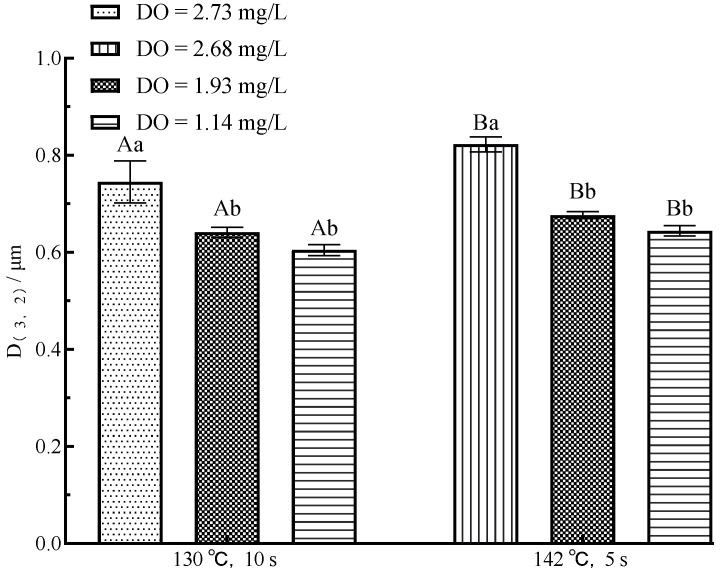
Effect of flash deoxygenation on particle size of UHT milk. Different uppercase letters indicate significant differences of each DO content among the UHT condition (*p* < 0.05). Different lowercase letters indicate significant differences among the DO content at each UHT condition (*p* < 0.05).

The typical particle size distribution of the sample is shown in Figure 3. The average milk particle size decreased when DO content decreased, inhibiting the aggregation of MFG and proteins. The effect of the DO content on the particle size of milk is primarily due to the contact of whey protein with DO to form a protein air/water interface, which then adsorbs fat globules [25]. The decrease in DO content limited the development of aggregates, resulting in a reduction in the particle size of the milk. However, the decrease in DO concentration in UHT milk with DO = 1.93 mg/L and DO = 1.14 mg/L under the same sterilization condition did not significantly change milk particle size (*p* > 0.05). The inability to further reduce the particle size may be related to the higher sterilization condition, and the UHT sterilization of milk increased the formation of sediment [26].

In addition to the effect of DO content variation in particle size, sterilization condition is also an important influencing factor. Under the same DO level, the particle size increased significantly with the increase in sterilization condition (*p* < 0.05), which was related to the increase in sterilization condition on accelerating fat floating and protein denaturation precipitation [27].

### 3.3. Effects of Flash Deoxygenation on the Oxidation of UHT Milk

The TOTOX value is an evaluation of both primary and secondary oxidation products and is used to estimate the oxidative deterioration of food lipids [28]. As shown in Table 2, the most significant effect on TOTOX values was observed when DO = 1.14 mg/L (*p* < 0.05). This is because DO plays a major role in the hyperoxidative phase of lipids, the fatty acid radical reacts with DO to form a peroxide free radical [29]. Therefore, the reduction in DO content in milk can inhibit lipid oxidation in UHT milk. On the other hand, the TOTOX value in deoxygenated UHT milk rose as the sterilization condition rose. The effect of sterilization conditions on lipid oxidation was more significant than deoxygenation. Furthermore, this is a secondary reason why the top TSI was smaller in the low DO content of UHT milk, since the reduction in lipid oxidation positively affected the milk fat stabilizer [30,31,32].

Protein oxidative status was evaluated as contents of PC and dityrosine. Significant differences were found in the PC content for the 4 kinds of UHT milk with different DO content (*p* < 0.05). Under the same UHT conditions, PC levels decreased significantly (*p* < 0.05) with decreasing DO content. Under the same DO content conditions, PC levels increased significantly with increasing sterilization conditions (*p* < 0.05). There were also differences between the 4 kinds of UHT milk with different DO content (*p* < 0.05) when the accumulation of dityrosine was measured by the oxidation method (Table 2). From Table 2, it can be seen that the content of dityrosine in UHT milk decreased when the DO content decreased. At 135 °C for 10 s, the content of dityrosine was reduced by approximately 47.95% when the DO content was reduced from 2.73 mg/L to 1.14 mg/L, and by approximately 32.12% at a sterilization condition of 145 °C for 5 s. Reduced PC and dityrosine levels are associated with lipid oxidation, which produces primary and secondary lipid oxidation products that react with proteins and cause protein oxidation [33]. The reduction in DO content inhibited lipid oxidation, decreasing the induction of protein oxidation by lipid oxidation [33]. In addition, flash deoxidation is also effective in reducing reactive oxygen species (ROS) in milk, thus reducing the occurrence of protein oxidation [34].

To sum up, reducing the DO content could indeed effectively reduce the oxidation of milk. However, the degree of oxidation of UHT milk increased with the increase in sterilization conditions under the same DO content.

### 3.4. Effects of Flash Deoxygenation on Lactulose in UHT Milk

**Figure 4:**
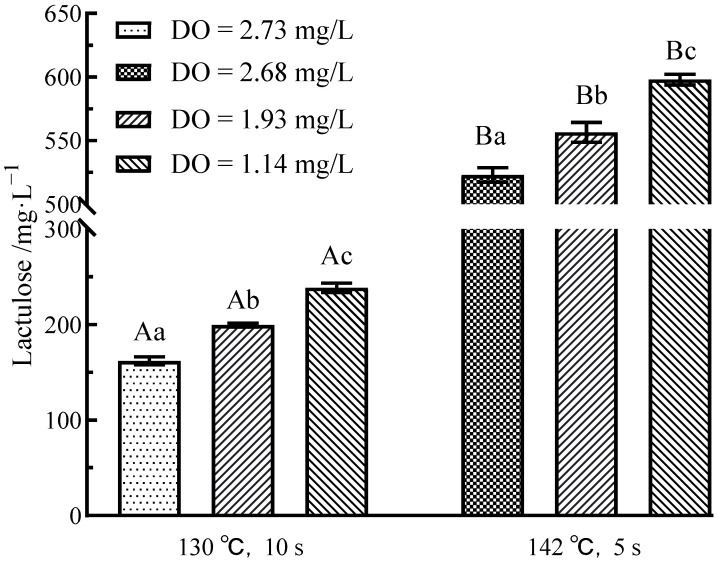
Effect of flash deoxygenation on lactulose content in UHT milk. Different uppercase letters indicate significant differences of each DO content among the UHT condition (*p* < 0.05). Different lowercase letters indicate significant differences among the DO content at each UHT condition (*p* < 0.05).

Lactulose is a disaccharide comprising fructose and forms due to an isomerization reaction of lactose during heating (>70 °C); it is also a participant in the early Maillard reaction [35]. According to Figure 4, under the same sterilization conditions, the lactulose content increased when the DO content decreased (*p* < 0.05). This is because the double bond of olefinic diol can be cleaved to form carboxylic acid under sufficient DO. The carboxylic acid will react with lactulose to form the early product of the Maillard reaction. Therefore, flash deoxidation treatment could remove DO to avoid the oxidative cleavage of the olefin diol, further preventing the consumption of lactulose [36]. However, the most critical factor affecting the lactulose content was the sterilization condition of the milk. Under higher sterilization conditions, the decrease in DO had no pronounced effect on the increase in lactulose content (*p* < 0.05).

### 3.5. Effects of Flash Deoxygenation on Furosine in UHT Milk

**Figure 5:**
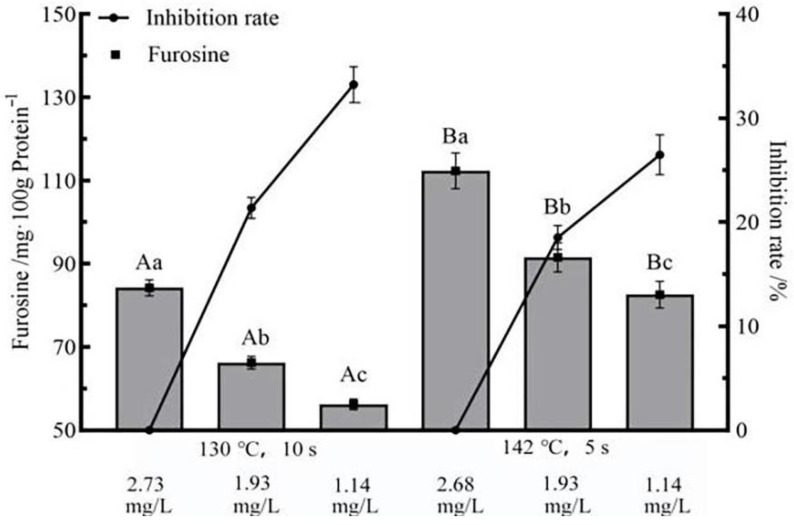
Effect of flash deoxygenation on the content of furosine in UHT milk. Different uppercase letters indicate significant differences of each DO content among the UHT condition (*p* < 0.05). Different lowercase letters indicate significant differences among the DO content at each UHT condition (*p* < 0.05).

The content of furosine is further indicative of changes in the early extent of the Maillard reaction. It can be seen from Figure 5 that when the content of DO decreased, the content of furosine in the milk also decreased. When DO = 1.14 mg/L, the average of furosine inhibition rate in milk with two different UHT sterilization conditions was 33.23 ± 1.72% and 26.48 ± 1.92%, respectively. When DO = 1.93 mg/L, the average of furosine inhibition rate in milk with two different UHT sterilization conditions was 21.38 ± 1.01% and 18.53 ± 1.16%, respectively. These results indicated that the content of the formation of furosine mainly depended on the heat treatment conditions of milk and the content of DO. Carboxylic acid (from olefinic diol double bond cleavage) and lactulose combine to form lactuloselysine, which is further hydrolyzed to furosine. The cleavage of olefinic diol was inhibited in UHT milk with low DO content, which inhibited the formation of lactulose lysine, thereby reducing the content of furosine in UHT milk [37]. Additionally, PC is one of the key conditions for the formation of furosine. The PC in milk decreased when the content of DO decreased, resulting in a decrease in the content of dicarbonyl compounds, thus inhibiting the production of furosine [38]. According to the decrease in DO content, the content of furosine in milk decreased. This result indicated that the decrease in DO content had an inhibitory effect on the early Maillard reaction. At the same time, it also showed that the reduction in DO inhibited the thermal damage of UHT milk.

### 3.6. Effects of Flash Deoxygenation on 5-HMF in UHT Milk

**Figure 6:**
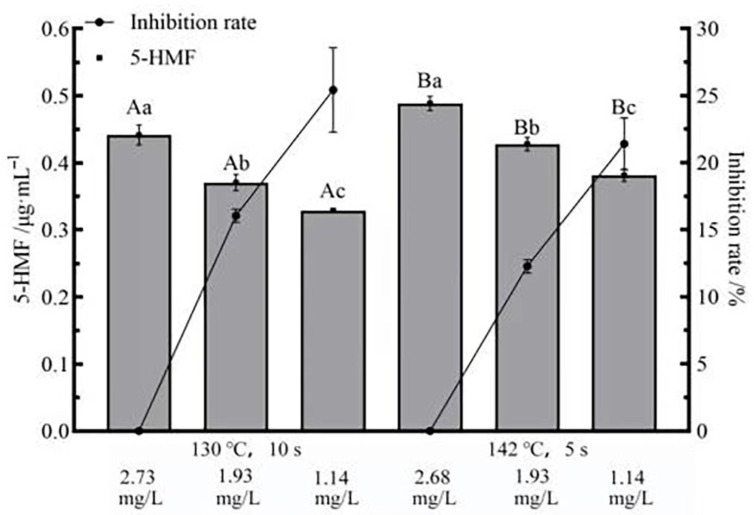
Effect of flash deoxidation on the content of 5-HMF in UHT milk. Different uppercase letters indicate significant differences of each DO content among the UHT condition (*p* < 0.05). Different lowercase letters indicate significant differences among the DO content at each UHT condition (*p* < 0.05).

5-HMF can be used to indicate the intermediate stage of the Maillard reaction. Figure 6 shows the effect of different DO contents on 5-HMF at two sterilization conditions. The average content of 5-HMF in the two undeoxygenated UHT milk was 0.442 ± 0.014 μg/mL and 0.488 ± 0.010 μg/mL. When DO = 1.14 mg/L, the average content of 5-HMF in milk with two different UHT sterilization conditions was 0.329 ± 0.003 μg/mL and 0.382 ± 0.009 μg/mL, and the average of 5-HMF inhibition was 25.43 ± 3.14% and 21.41 ± 1.93%. When DO = 1.93 mg/L, the average content of 5-HMF in milk with two different UHT sterilization conditions was 0.384 ± 0.017 μg/mL and 0.407 ± 0.008 μg/mL, and the average of 5-HMF inhibition was 16.04 ± 0.49% and 12.29 ± 0.49%. 5-HMF concentration reduced with the decrease in DO concentration (*p* < 0.05). The decrease in 5-HMF content may have the following 3 reasons. (a) The reduction in DO content inhibited the degradation reaction rate of olefinic diols, thereby reducing the content of 5-HMF [39]. (b) Lower DO content inhibited the formation of 5-HMF by inhibiting the dicarbonyl compounds derived from the PC [40,41]. (c) A lower DO level prevented the oxidation of ascorbic acid from generating furfural compounds, inhibiting the formation of 5-HMF [4]. In addition, the increase in sterilization conditions promoted the formation of 5-HMF, which was consistent with the previous findings that the increase in heat treatment temperature promoted the formation of 5-HMF [39].

### 3.7. Effects of Flash Deoxygenation on Melanoids in UHT Milk

**Figure 7:**
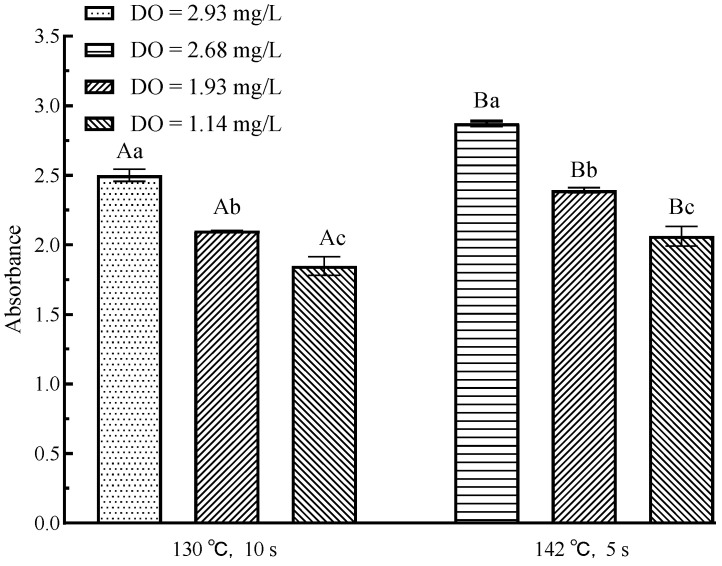
Changes in melanoids in UHT milk by flash deoxygenation. Different uppercase letters indicate significant differences of each DO content among the UHT condition (*p* < 0.05). Different lowercase letters indicate significant differences among the DO content at each UHT condition (*p* < 0.05).

Melanoids are the final product of the Maillard reaction, and their absorbance change can be used as an indicator of the late Maillard reaction. Figure 7 showed the change in absorbance of melanoids in milk after flash deoxygenation. Regardless of the UHT sterilization conditions, the absorbance of deoxygenated UHT milk decreased significantly compared to that of un-deoxygenated samples (*p* < 0.05), indicating a decrease in melanoids content. The content of melanoids in UHT milk decreased significantly with decreasing DO content (*p* < 0.05). The inhibition of melanoids by the DO content is mainly related to lipid oxidation and inhibition of 5-HMF. DO oxidizes fatty acids (especially unsaturated fatty acids) to aldehydes and ketones, which subsequently combine with amino acids to form melanoids. In addition, the browning reaction of Amadori products was induced by the peroxidation product (lipid oxidation) to produce melanoids [42,43]. At the end of the Maillard reaction, 5-HMF polymerizes with aldehydes and ketones obtained from the melatonin reaction or lipid oxidation to further produce melanoids [44]. Therefore, a decrease in DO content inhibited the formation of 5-HMF and lipid oxidation, which in turn inhibited the formation of melanoids [45]. With the same DO content, the absorbance of UHT milk sterilized at 145 °C for 5 s was significantly greater (*p* < 0.05), indicating that the increase in sterilization condition led to an increase in the content of Melanoids in milk, which was consistent with the change in 5-HMF.

### 3.8. Effect of Flash Deoxygenation on the Color of UHT Milk

Color is also an important indicator for evaluating the quality of UHT milk. Results (Table 3) showed that the effects of DO content and sterilization condition on browning were opposite, and the reduction in DO content inhibited browning. Deoxygenated UHT milk (L*) increased with the decrease in DO content due to the suppressed Maillard reaction. Contrarily, values of a*, b* and BI decreased with decreasing DO content, denoting the decline in red and yellow characteristics in the system. Sterilization had the opposite effect on the color of UHT milk. The effect of the reduction in DO content on the browning of UHT milk was mainly due to the following reasons. (a) The reduction in DO content inhibited the Maillard reaction and reduced the content of melanoid, thereby reducing the degree of browning of milk. (b) The free radicals generated by lipid oxidation led to the browning of lactose in milk. After deoxygenation, the lipid oxidation in milk was inhibited, thereby reducing the browning of lactose [46]. In addition, lipid oxidation can participate in the Maillard reaction, contributing to non-enzymatic browning [43]. (c) Strecker aldehydes can condense with carbonyl compounds forming brown pigments and affecting the color as well [47,48]. The reduction in DO content inhibited protein oxidation and reduced the effect of carbonyl compounds on color.

### 3.9. Effects of Flash Deoxygenation on the Content of Volatile Flavor Substances of UHT Milk

UHT sterilization affects not only the lipids, proteins, and lactose in milk but also its volatile flavor substances. There are some peculiar smells in the milk after UHT sterilization, mainly including “oxidation”, “stale”, and “cooking” [49]. As shown in Table 4, the content of some special volatile flavor substances in UHT milk after flash deoxygenation was changed. The contents of ketones and aldehydes in UHT milk after deoxygenation were significantly decreased (*p* < 0.05). This is because large amounts of ketones and aldehydes are produced during lipid oxidation and Maillard reactions. After flash deoxygenation, lipid oxidation and Maillard reactions in UHT milk were inhibited, thus reducing the content of these ketones and aldehydes. These formed aldehydes and ketones adversely affect the flavor of milk, such as 2-heptanone and 2-nonanone in ketones. When 2-heptanone or 2-nonanone alone appears as a fruity odor, they will bring a “stale” smell to the milk when the two exist simultaneously [50]. In addition, aldehydes are also the main contributors to the “stale” flavor in UHT milk, of which octanal, nonanal, and decanal are the main contributors to the “stale” flavor. The remaining ketones (2-pentanone, 2-hexanone, 2-octanone, 2-decanone, and 2-undecanone) and aldehydes (valeraldehyde, hexanal, heptanal, octanal, nonanal and decanal) contribute to the oxidative odor of UHT. The experimental results showed that the deoxygenation treatment had a positive effect on the volatile flavor substances of UHT milk.

However, the effect of decreasing DO content on sulfide was not apparent (*p* > 0.05). Sulfide is the source of cooking flavor in UHT milk and is formed by protein denaturation. The reduction in DO content did not affect the deformation of the protein (especially whey protein) [51] and, thus, could not effectively inhibit the formation of sulfides. The slight decrease in sulfide content may be related to lipid oxidation and Maillard reaction [52,53]. A small amount of sulfide comes from lipid and protein oxidation. It may be because lipid oxidation and Maillard reaction were inhibited by DO content, resulting in a decrease in sulfide levels.

## 4. Conclusions

This study comprehensively analyzed the UHT milk quality associated with different DO contents such as preprocessed using a flash deoxygenation technique. The UHT milk with DO = 1.14 was found to be the most stable and least oxidized under 135 °C for 10 s sterilization. Particle size, TOTOX value, PC value, and Dityrosine were the smallest, with 0.605 ± 0.011 μm, 5.02 ± 0.06, 1.65 ± 0.11 nmol/mg protein, and 0.61 ± 0.12 AFU/mg protein, respectively. At this time, the inhibition rate of furosine could reach 33.23 ± 1.72%, and the inhibition rate of 5-HMF was 25.43 ± 3.14%, while the least volatile substances with undesirable properties were contained.

From the perspective of UHT milk stability, the changes in the lipid and protein stability in UHT milk depended on the intensity of both the DO content and sterilization condition. Lipid and protein stability increased with decreasing DO content or sterilization condition. From Oxidation, Maillard reaction, color, and flavor analysis, these reactions affected each other. The effect of DO on these reactions first acted on oxidation. The reduction in DO content inhibited lipid and protein oxidation in UHT and the other three reactions by reducing oxidation products. In dairy production, the reduction in DO content helps maintain the stability of UHT milk and positively impacts its quality.

These data provide important insights into changes in the variation of DO content on the quality of different UHT sterilized milk and establish a new production method for future studies that can be extended to the production of pasteurized and ultra-pasteurized milk to improve their quality.

## Figures and Tables

**Table 1:** Contents of milk components after flash evaporation.

Flash Condition	UHT Condition	Fat (%)	Protein (%)	Lactose (%)	Dry Matter (%)	DO (mg/L)
-	-	2.85 ± 0.32 ^a^	3.03 ± 0.17 ^a^	4.05 ± 0.22 ^a^	11.24 ± 0.42 ^a^	2.93 ± 0.35 ^a^
-	135 °C, 10 s	3.12 ± 0.15 ^a^	3.02 ± 0.09 ^a^	4.27 ± 0.12 ^a^	11.47 ± 0.23 ^a^	2.73 ± 0.27 ^b^
65 °C,−0.08 MPa	135 °C, 10 s	3.75 ± 0.14 ^b^	3.36 ± 0.10 ^b^	4.77 ± 0.15 ^b^	12.16 ± 0.11 ^b^	1.14 ± 0.15 ^c^
70 °C,−0.06 MPa	135 °C, 10 s	3.59 ± 0.18 ^b^	3.33 ± 0.12 ^b^	4.80 ± 0.17 ^b^	12.12 ± 0.17 ^b^	1.93 ± 0.23 ^d^
-	145 °C, 5 s	2.77 ± 0.23 ^a^	3.05 ± 0.11 ^a^	4.22 ± 0.21 ^a^	11.34 ± 0.23 ^a^	2.68 ± 0.29 ^b^
65 °C,−0.08 MPa	145 °C, 5 s	3.79 ± 0.21 ^b^	3.42 ± 0.15 ^b^	4.72 ± 0.23 ^b^	12.19 ± 0.21 ^b^	1.14 ± 0.18 ^c^
70 °C,−0.06 MPa	145 °C, 5 s	3.63 ± 0.24 ^b^	3.41 ± 0.20 ^b^	4.75 ± 0.24 ^b^	12.05 ± 0.27 ^b^	1.93 ± 0.27 ^d^

Note: Different lowercase letters in the same column indicate significant difference between treatments (*p* < 0.05).

**Table 2:** Changes in oxidation in UHT milk after flash deoxygenation.

UHT Condition	Flash Condition	DO (mg/L)	TOTOX Value	PC (nmol/mg of Protein)	Dityrosine (AFU/mg of Protein)
135 °C, 10 s	-	2.73	6.73 ± 0.21 ^Aa^	3.17 ± 0.12 ^Aa^	0.84 ± 0.15 ^Aa^
	70 °C, −0.06 MPa	1.93	5.86 ± 0.11 ^Ab^	2.32 ± 0.07 ^Ab^	0.73 ± 0.13 ^Ab^
	65 °C, −0.08 MPa	1.14	5.02 ± 0.06 ^Ac^	1.65 ± 0.11 ^Ac^	0.61 ± 0.12 ^Ac^
145 °C, 5 s	-	2.68	7.47 ± 0.25 ^Ba^	3.58 ± 0.06 ^Ba^	1.06 ± 0.10 ^Ba^
	70 °C, −0.06 MPa	1.93	6.81 ± 0.13 ^Bb^	2.99 ± 0.09 ^Bb^	0.93 ± 0.15 ^Bb^
	65 °C, −0.08 MPa	1.14	6.07 ± 0.18 ^Bc^	2.43 ± 0.13 ^Bc^	0.81 ± 0.14 ^Bc^

Note: Different uppercase letters in the same column indicate significant differences of each DO content among the UHT condition (*p* < 0.05). Different lowercase letters in the same column indicate significant differences among the DO content at each UHT condition (*p* < 0.05).

**Table 3:** Color changes in UHT milk after flash deoxygenation.

UHT Condition	Flash Condition	DO (mg/L)	L*	a*	b*	*BI*
135 °C, 10 s	-	2.73	82.97 ± 0.52 ^Aa^	9.52 ± 0.66 ^Aa^	22.01 ± 0.22 ^Aa^	69.23 ± 0.14 ^Aa^
	70 °C, −0.06 MPa	1.93	85.78 ± 0.27 ^Ab^	8.33 ± 0.10 ^Ab^	20.67 ± 0.11 ^Ab^	62.28 ± 0.26 ^Ab^
	65 °C, −0.08 MPa	1.14	89.12 ± 0.49 ^Ac^	7.42 ± 0.22 ^Ac^	18.34 ± 0.36 ^Ac^	53.71 ± 0.19 ^Ac^
145 °C, 5 s	-	2.68	77.13 ± 0.04 ^Ba^	10.15 ± 0.12 ^Ba^	25.32 ± 0.11 ^Ba^	72.79 ± 0.25 ^Ba^
	70 °C, −0.06 MPa	1.93	83.42 ± 0.26 ^Bb^	9.17 ± 0.12 ^Bb^	23.82 ± 0.15 ^Bb^	67.56 ± 0.21 ^Bb^
	65 °C, −0.08 MPa	1.14	87.69 ± 0.59 ^Bc^	8.37 ± 0.19 ^Bc^	20.25 ± 0.42 ^Bc^	60.25 ± 0.29 ^Bc^

Note: Different uppercase letters in the same column indicate significant differences of each DO content among the UHT condition (*p* < 0.05). Different lowercase letters in the same column indicate significant differences among the DO content at each UHT condition (*p* < 0.05).

**Table 4:** Effects of concentration (μg/kg) of certain flavor compounds in UHT milk after flash deoxygenation.

Compound	135 °C, 10 s			145 °C, 5 s		
	DO = 2.73 mg/L	DO = 1.93 mg/L	DO = 1.14 mg/L	DO = 2.68 mg/L	DO = 1.93 mg/L	DO = 1.14 mg/L
**Ketones**						
2-Pentanone	6.59 ± 0.34 ^Aa^	5.65 ± 0.22 ^Ab^	4.83 ± 0.21 ^Ac^	6.97 ± 0.21 ^Ba^	6.30 ± 0.25 ^Bb^	5.59 ± 0.24 ^Bc^
2-Hexanone	1.23 ± 0.11 ^Aa^	0.75 ± 0.08 ^Ab^	0.54 ± 0.09 ^Ac^	1.39 ± 0.13 ^Ba^	0.86 ± 0.15 ^Bb^	0.67 ± 0.09 ^Bc^
2-Heptanone	43.01 ± 3.41 ^Aa^	39.43 ± 3.55 ^Ab^	37.43 ± 2.34 ^Ac^	46.69 ± 3.51 ^Ba^	43.33 ± 2.61 ^Bb^	40.69 ± 2.23 ^Bc^
2-Octanone	4.51 ± 0.18 ^Aa^	3.78 ± 0.15 ^Ab^	3.24 ± 0.10 ^Ac^	4.87 ± 0.16 ^Ba^	4.28 ± 0.13 ^Bb^	3.64 ± 0.16 ^Bc^
2-Nonanone	4.05 ± 0.22 ^Aa^	3.37 ± 0.13 ^Ab^	2.78 ± 0.19 ^Ac^	4.43 ± 0.22 ^Ba^	3.81 ± 0.17 ^Bb^	3.13 ± 0.13 ^Bc^
2-Decanone	1.45 ± 0.17 ^Aa^	1.06 ± 0.12 ^Ab^	0.78 ± 0.11 ^Ac^	1.72 ± 0.22 ^Ba^	1.36 ± 0.18 ^Bb^	0.98 ± 0.15 ^Bc^
2-Undecanone	9.99 ± 0.11 ^Aa^	9.38 ± 0.14 ^Ab^	8.74 ± 0.15 ^Ac^	10.33 ± 0.10 ^Ba^	9.62 ± 0.18 ^Bb^	9.03 ± 0.12 ^Bc^
**Aldehydes**						
Valeraldehyde	0.69 ± 0.15 ^Aa^	0.42 ± 0.13 ^Ab^	0.35 ± 0.14 ^Ac^	0.79 ± 0.16 ^Ba^	0.62 ± 0.17 ^Bb^	0.55 ± 0.15 ^Bc^
Hexanal	12.31 ± 1.62 ^Aa^	10.27 ± 1.26 ^Ab^	9.18 ± 1.05 ^Ac^	13.49 ± 1.12 ^Ba^	11.22 ± 1.37 ^Bb^	10.38 ± 1.35 ^Bc^
Heptanal	5.71 ± 0.59 ^Aa^	4.61 ± 0.39 ^Ab^	3.79 ± 0.68 ^Ac^	6.54 ± 0.48 ^Ba^	5.79 ± 0.65 ^Bb^	4.83 ± 0.44 ^Bc^
Octanal	0.92 ± 0.11 ^Aa^	0.76 ± 0.14 ^Ab^	0.56 ± 0.07 ^Ac^	1.15 ± 0.12 ^Ba^	0.92 ± 0.12 ^Bb^	0.75 ± 0.14 ^Bc^
Nonanal	3.76 ± 0.31 ^Aa^	2.47 ± 0.25 ^Ab^	1.75 ± 0.16 ^Ac^	3.96 ± 0.37 ^Ba^	2.72 ± 0.27 ^Bb^	1.96 ± 0.25 ^Bc^
Decanal	6.63 ± 0.25 ^Aa^	5.51 ± 0.18 ^Ab^	4.42 ± 0.21 ^Ac^	6.97 ± 0.36 ^Ba^	5.83 ± 0.27 ^Bb^	4.88 ± 0.22 ^Bc^
**Sulphur compounds**						
Hydrogen sulfide	11.83 ± 1.83 ^Aa^	10.79 ± 1.52 ^Aa^	9.31 ± 1.39 ^Aa^	12.46 ± 1.77 ^Aa^	11.59 ± 1.48 ^Aa^	10.21 ± 1.65 ^Aa^
Methyl mercaptan	23.17 ± 2.50 ^Aa^	22.03 ± 2.42 ^Aa^	21.93 ± 2.39 ^Aa^	25.53 ± 2.32 ^Aa^	24.33 ± 2.41 ^Aa^	23.23 ± 2.61 ^Aa^
Methyl sulfide	21.32 ± 1.92 ^Aa^	19.98 ± 2.13 ^Aa^	19.17 ± 1.72 ^Aa^	24.52 ± 2.88 ^Aa^	23.95 ± 1.76 ^Aa^	23.27 ± 1.73 ^Aa^
Dimethyl sulfoxide	1.43 ± 0.12 ^Aa^	1.28 ± 0.11 ^Aa^	1.11 ± 0.15 ^Aa^	1.53 ± 0.22 ^Aa^	1.41 ± 0.21 ^Aa^	1.29 ± 0.14 ^Aa^

Note: Different uppercase letters in the same column indicate significant differences of each DO content among the UHT condition (*p* < 0.05). Different lowercase letters in the same column indicate significant differences among the DO content at each UHT condition (*p* < 0.05).

## Data Availability

The data presented in this study are available on request from the corresponding author.
